# IgA B Cell Responses to Gut Mucosal Antigens: Do We Know it all?

**DOI:** 10.3389/fimmu.2013.00368

**Published:** 2013-11-13

**Authors:** Nils Y. Lycke

**Affiliations:** ^1^Department of Microbiology and Immunology, Institute of Biomedicine, University of Gothenburg, Gothenburg, Sweden

**Keywords:** IgA, intestinal, germinal centers, Peyer’s patches, gut lamina propria

In this Research Topic of Frontiers in Mucosal Immunology we have focused on gut IgA B cell responses. We have invited world leading researchers in the field to contribute with articles that give the most recent up-date on our current understanding of this interesting and complex protective system. Because a majority of all human pathogens access the body via the mucosal membranes we need an effective innate and adaptive immune system to protect against infectious diseases at these sites. Secretory IgA (SIgA) antibody formation is a hallmark of this protective barrier function (1). However, it does not only play a role for protection against pathogens, it also serves as a key element in establishing stable homeostasis between the host and the microbiota of the mucosal membranes (2). For example, the gut IgA system develops after weaning in early life and critically influences the symbiosis between the host and the commensal bacteria (3, 4). Recent data suggest that each individual accumulates highly mutated gut IgA gene sequences over time and that the development of long-term memory B cells is a cardinal feature of mucosal IgA immunity (5). This appears to apply not only to B cells responding to T cell-dependent (TD) antigens, but, in fact, may also apply to B cells responding to T cell-independent (TI) antigens derived from the microbiota (6). To explain the high level of mutations in gut IgA gene sequences in adults it has been proposed that the IgA B cell inductive sites, primarily the Peyer’s patches (PP), play a major role in allowing extensive cell division and, hence, promoting multiple mutations (7). Since mutations are acquired in germinal centers and these are formed as a consequence of T-B cell cooperation following antigen stimulation it has become increasingly important to understand the fine detail of how germinal centers in the PP are regulated. A prime question is whether germinal centers in PP also could host B cells that respond to TI-antigens, which could then promote cell division and allow for the accumulation of multiple mutations in differentiating B cells. Recent findings suggest that activated gut B cells can re-utilize already established germinal centers in PP and in this way gut SIgA responses can be synchronized and oligoclonal, securing a high quality of the protective SIgA antibodies in the lamina propria (8). Based on these observations the function of follicular helper T cells (T_FH_) in PP then appear to be quite unique compared to that found in peripheral lymph nodes. In fact, failure to develop appropriate T_FH_ (PD-1 deficiency) in PP have been found to drastically affect the composition of the microbiota, arguing in favor of that IgA B cell differentiation directly impacts on gut homeostasis and the presence of certain bacterial species (9). Mice that lack the ability to mutate IgA B cell responses have been shown to host bacterial overgrowth in the gut and have a dysfunctional mucosal barrier in the absence of IgA affinity maturation (10). Importantly, erroneous activation of the mucosal immune system can result in chronic inflammation and development of autoimmunity or allergy. Thus, non-responsiveness or immune tolerance, is critical and a special feature of the regulatory mechanisms that prevail at mucosal immune inductive sites (11). Hence the mucosal immune system must maintain a finely tuned balance between tolerance and responsiveness to mucosal antigens, including TI-antigens derived from the microbiota. As already discussed, this does not mean that the gut SIgA system is ignorant about the microbiota, but rather the contrary, that there is a balance in the adaptive immune response to establish homeostasis and avoid inflammatory reactions.

A series of review articles, which broadly address the different aspects of SIgA and how gut intestinal B cell responses are generated and regulated, is presented in this Research Topic. The first article by Knoop and Newberry gives an overview of the gut mucosal IgA-inductive sites and discusses the role of the various inductive tissues; PP, isolated lymphoid follicles (ILF), and colon patches for normal SIgA homeostasis and what happens in immune dysfunction. The second review paper by Kunisawa and Kiyono focuses on the PP as inductive site for SIgA responses and describes how commensal bacteria can stimulate SIgA, which then can promote increased up-take of bacteria into the PP tissue and also how this mechanism can establish intra-PP habitation of *Alcaligenes* bacteria, i.e., what are the consequences of having an indigenous commensal in PP. Slack, Balmer, Fritz, and Hapfelmeier then describes in the third review the functional flexibility of intestinal IgA formation that is also key to understanding how symbiosis with the microbiota can be maintained in the gut. This is also a theme entertained by Corthésy as he describes the multi-faceted functions of SIgA in the fourth review paper. Special attention is given to the innate properties of SIgA as an anti-inflammatory regulator, showing that not only Fab-specific antigen-recognition contributes to gut homeostasis and protection against pathogens, but also that SIgA exerts an effect on epithelial cell integrity and the mucosal barrier functions. The fifth review paper in this Research Topic explores the different findings of enhanced IgA plasma cell activity in gut lamina propria of patients with celiac disease, the prototypic gut inflammatory condition that results from a dysfunctional immune response to dietary gluten. Especially, Mesin, Sollid, and Di Niro describes long-term plasma cell production of autoantibodies in the lamina propria against transglutaminase 2, which may constitute as many as 4–24% of the total IgA plasma cell population in celiac lesions. Spencer, Klavinskis, and Fraser continuous on this line of human immunobiology of the SIgA system and discuss burning questions relating to how to understand its organization and regulation in health and disease. In particular, comparisons are made between what we know about human SIgA immune responses and the information we have gained from experimental rodent models. The next review article by Lycke and Bemark addresses how we can explain the fact that SIgA immune responses to TD-antigens in the gut are oligoclonal and appear to be synchronized along the entire small and large intestine. The role of PP in this context is highlighted and regulatory aspects of PP germinal centers are presented in a challenging theory. Finally, the contribution by Conner and Blutt describes various functional aspects of gut SIgA responses against gastrointestinal virus infections and discusses what we can do to harness the protective properties of SIgA to achieve better mucosal vaccines.
